# Optimal Rate Schedules with Data Sharing in Energy Harvesting Communication Systems

**DOI:** 10.3390/s17122958

**Published:** 2017-12-20

**Authors:** Weiwei Wu, Huafan Li, Feng Shan, Yingchao Zhao

**Affiliations:** 1 School of Computer Science and Engineering, Southeast University, Nanjing 210018, China; weiweiwu@seu.edu.cn (W.W.); huafan@seu.edu.cn (H.L.); 2 School of Computing and Information Sciences, Caritas Institute of Higher Education, Hong Kong 999077, China; zhaoyingchao@gmail.com

**Keywords:** wireless data transmission, rate schedule, data sharing, energy-efficiency, energy harvesting, algorithm design

## Abstract

Despite the abundant research on energy-efficient rate scheduling polices in energy harvesting communication systems, few works have exploited data sharing among multiple applications to further enhance the energy utilization efficiency, considering that the harvested energy from environments is limited and unstable. In this paper, to overcome the energy shortage of wireless devices at transmitting data to a platform running multiple applications/requesters, we design rate scheduling policies to respond to data requests as soon as possible by encouraging data sharing among data requests and reducing the redundancy. We formulate the problem as a transmission completion time minimization problem under constraints of dynamical data requests and energy arrivals. We develop offline and online algorithms to solve this problem. For the offline setting, we discover the relationship between two problems: the completion time minimization problem and the energy consumption minimization problem with a given completion time. We first derive the optimal algorithm for the min-energy problem and then adopt it as a building block to compute the optimal solution for the min-completion-time problem. For the online setting without future information, we develop an event-driven online algorithm to complete the transmission as soon as possible. Simulation results validate the efficiency of the proposed algorithm.

## 1. Introduction

Energy harvesting from environments has been explored and implemented as an alternative to supplement or even replace batteries in modern wireless communication systems [1]. In such systems, energy harvesting techniques enable wireless devices to prolong the lifetime of operating by accumulating energy from surrounding light, thermal and kinetic energy, etc. [2,3]. Meanwhile, more and more wireless devices nowadays are capable of adaptively changing the transmission power or rate for the purpose of improving energy efficiency [4]. As it is widely known, the relationship between the rate and power follows a convex function by the nature of encoding schemes [5]. Thus, although the energy harvesting technique has potential of improving the power supply in the long term, we still have to carefully design energy-efficient rate scheduling policies, considering that the harvested energy is usually limited and unstable in the short-term.

Although there have been many research efforts on designing rate scheduling algorithms in energy harvesting communication systems, most of the previous works model the transmission in an isolated and passive manner. In other words, the job of the transmitter is to try its best to deliver requested data (packets) exactly as accumulated in the buffer. However, in some scenarios, requested data sending to the remote control platform can be shared. In such a scenario, different applications on the platform may generate different requests of data based on their own need. Although data requests arrive at the transmitter at different time and in different required amount, the transmitted data can be shared by multiple requesters or applications, which would further save energy as well as data traffic [6,7]. For example, in traffic monitoring sensor network systems, there are many applications in the control center platform, such as driving directions computation, traffic characterization, congestion prediction, cab fleet management or urban planning tools [8]. These applications may request remote data, such as volume and average speed of traffic sampled, from a road traffic sensor. These information may be requested (in different time and amount) by different applications, therefore, the road traffic sensor can combine and share data transmission among data requests. Besides, in participatory sensing systems, data sensed from smartphones should be transmitted back to the centric platform, and the sensed data may be shared/requested by multiple applications [9].

**Figure 1:**
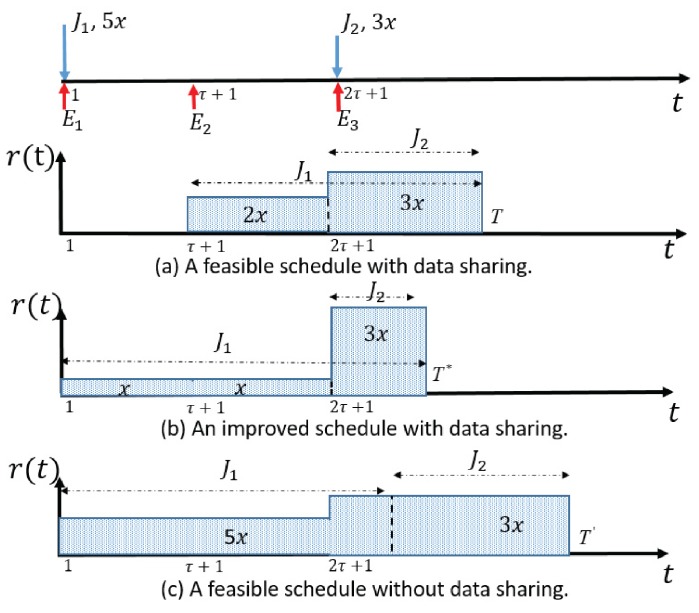
Exampary schedules with and without data sharing.

We use an example in Figure 1 to further illustrate the core idea and its efficiency of data sharing. In this example, there are three energy harvestings with arrival time 1,τ+1 and 2τ+1. There are two data requests $J_{1},J_{2}$ that request an amount 5x of sensed data after time 1 and an amount 3x of sensed data after time 2τ+1, respectively. The transmitter needs to transmit the required data to the platform/receiver with minimum completion time while satisfying the data requests without violating energy constraints. Figure 1a shows a feasible schedule that completes the transmission at time *T* by sharing the sensed data between requests $J_{2}$ and $J_{1}$. That is, the sensed data with amount of 3x in interval [2τ+1,T] is transmitted and shared with both requests. Now request $J_{2}$ is already fully satisfied. The remaining data amount of request $J_{1}$ is satisfied by transmitting 2x sensed data in time interval [τ+1,2τ]. Although this schedule reduces the completion time by exploiting data sharing, it is not the optimal one. Figure 1b illustrates a better schedule with a shorter completion time $T^{*}$, which transmits the 2x amount of data equally in interval [1,2τ]. Such a new schedule saves the energy consumed in interval [1,2τ] (due to the convexity of the rate-power function) and allows more energy to complete the transmission of the rest data in a shorter time. Figure 1c demonstrates that if data sharing is not exploited, the resulting competition time will be much longer since the sensed data is transmitted in an isolated manner to satisfy the requests separately.

In this paper, we investigate the optimal rate scheduling policies by exploiting the data sharing for energy harvesting communication devices so as to transmit the required data of requests with the minimum completion time. The proposed rate schedule policy must (1) consume no more energy than the accumulated energy by any time slot and efficiently utilize the energy, (2) share the data as much as possible and fulfill the data requirement, (3) complete transmission as soon as possible. According to the knowledge of the authors, all previously designed policies in the literature either have not exploited data sharing or can only work when all energy are available at the beginning, thus are not applicable to energy harvesting communication systems with the consideration of data sharing. A full review can be referred to in Section 2.

Our contributions are summarized as follows.

- This paper introduces a rate scheduling problem for energy harvesting wireless devices that transmit required data of requests with the goal of minimizing the completion time. We exploit the data sharing among data requests from the platform, e.g., a participatory sensing system, to actively enhance the energy utilization of the wireless device.
- We first study a closely related min-energy problem that aims to minimize the energy consumption within a given deadline while transmitting all required data. By decomposing the original problem into two simplified known sub-problems, we derive the optimal offline algorithm Bottleneck-Select  that minimizes the energy consumption or determines that no feasible solution exists within the given deadline.
- Then, by adopting Bottleneck-Select as a building block, we develop an optimal offline algorithm for the completion time minimization problem. The idea is to use Bottleneck-Select to narrow down the lower bound and upper bound of the minimum completion time, and then precisely locate the optimal solution.
- We also design an event-driven online heuristic algorithm to deal with the dynamic energy and request arrivals. Simulation results validate that its performance is close to the optimal offline solution.

The rest of this paper is organized as follows. We provide an overview of related work in Section 2. In Section 3, we define the system model and formulate the optimization problems. Section 4 provides the optimal algorithm to minimize the energy consumption and determine the existence of feasible solution within a given deadline. In Section 5, we first discuss the relationship between the min-energy problem and two known simplified models, and then derive the optimal algorithm for the completion time minimization problem. Online algorithm and simulations are presented in Section 6 and Section 7, respectively. Finally, we conclude the paper in Section 8.

## 2. Related Work

### 2.1. Rate-Adaptive Transmission

The design of rate-adaptive transmission algorithms with energy harvesting consideration have been widely studied. There are mainly two types of scenarios in the literature, e.g., pre-arrived data transmission [10,11,12,13,14] and dynamically arriving packet transmission [15,16,17,18,19,20,21,22].

The pre-arrived data transmission scheduling assumes unlimited data to be delivered to investigate the wireless channel capacity and throughput. Gatzianas et al. [10] explore the rate transmission problem with the objective of maximizing total system utility for an energy harvesting sensor node from a stochastic aspect by developing a queue stabilizing policy. Sharma et al. [11] study the energy management policies for throughput maximization in an energy harvesting sensor node. Vaze et al. [12] propose a competitive online algorithm that achieves a throughput within a bounded factor of the optimal throughput. Wu et al. [13] further consider the battery overflow in developing online algorithms with bounded competitive ratios to the maximum throughput. Xu and Zhang [14] address the problem of characterizing the fundamental trade-off of maximizing energy efficiency versus spectrum efficiency in a point-to-point AWGN channel.

The dynamically arriving packet transmission scheduling assume a group of packets to be delivered. Yang et al. are among the first group to develop packet transmission policies that take into account the dynamic arrivals of data packets in energy harvesting communication systems [15,16]. Since then, a series of works have investigated the rate transmission policies with packets/tasks consideration. There are two major goals, e.g., minimizing the transmission completion time and minimizing the energy consumption. Chen et al. [17,18,19] investigate the rate scheduling policies that transmit data packets and meet the delay constraints in static channels. Shan et al. [20] study the same problem by further assuming the allowable rate is discrete. Ozel et al. [21] develop rate schedules with the aim of minimizing the transmission completion time in sending a given packet in a wireless fading channel. Shan et al. [20] consider the problem of minimizing the energy consumption for dynamically arrived packets with individual deadlines. Deshmukh and Vaze [22] target at designing online algorithms that use minimum energy to transmit a set of dynamically arriving packets within given deadlines.

### 2.2. Data Sharing

In [8], Tavakoli et al. first formulate the data sharing problem and develop online methods to detect when to share and how to eliminate redundancies. Fang et al. [23] then introduce the interval data sharing problem, which aims to transmit as less data as possible while guaranteeing the QoS constraints of all applications. Zhao et al. [24] consider the fairness among users when scheduling tasks by optimizing the min-max aggregate sensing time of the users. Zhao et al. [25] assume the data sampling is continuous and propose a 2-approximate algorithm for maximizing the data sharing.

Wu et al. [9,26], further point out that besides the trade-off between energy consumption and QoS constraints, there also exists a trade-off between the transmission redundancy and energy consumption in a communication system consisting of rate-adaptive wireless devices. In their research, they formulate the problem as a bi-objective optimization problem and develop competitive online algorithms to simultaneously minimize the data traffic and the energy consumption, which is the most relevant one to the present work. Although data sharing is incorporated to actively enhance the energy usage, all previously designed policies can only work when all energy are available at the beginning, thus are not applicable to energy harvesting communication systems with dynamical arrivals of energy.

## 3. Preliminaries

In this section, we first introduce the system model of energy-efficient data transmission with energy harvesting and data sharing, and then formulate the problem.

### 3.1. System Model

We consider an energy harvesting wireless communication system where a wireless transmitter needs to transmit sensed data to a platform as requested.

The system time is equally partitioned into time slots with unit length, labeled as slot 1,2,…. We assume a time slot is the shortest time unit at which we apply a schedule, and the rate/power in one time slot is constant.

We model data requests of applications as tasks. Let $J=\{J_{1},J_{2},\ldots ,J_{n}\}$ be a set of *n* tasks to be accomplished where each task/request $J_{i}$ is represented as a pair $\left(a_{i},w_{i}\right)$, which means an amount $w_{i}$ of sensed data after time $a_{i}$ is requested by $J_{i}$. $a_{i}$ is called an *arrival point*. Without loss of generality, we assume $1=a_{1}<a_{2}<\ldots <a_{n}$.

We assume data sharing among tasks/requests, where each request has a specified time requirements of sensed data, and the sensed data in overlapped time period can be shared by two requests, following the same data sharing model in [8,9,26].

Let $H=\{H_{1},H_{2},\ldots ,H_{m}\}$ be a set of *m* energy harvesting events, where $H_{i}=\left(c_{i},E_{i}\right)$ means that $E_{i}$ amount of energy is harvested in time slot $c_{i}$ by the transmitter. We assume that the $E_{i}$ amount of energy can be immediately used at the beginning of the time slot $c_{i}$. For each harvesting $H_{i}=\left(c_{i},E_{i}\right)\;\left(1\leq i\leq m\right)$, we say that a harvesting event occurs at time $c_{i}$ and $c_{i}$ is called a *harvesting point*.  Without loss of generality, we assume $1=c_{1}<c_{2}<\ldots <c_{m}$. By incorporating the initial energy in the battery into the first harvesting event $H_{1}$, we treat the battery empty at the very beginning.

Obviously, there are totally (m+n) *event points*, $e_{i},i=1,2,\ldots ,m+n$ and $1=e_{1}\leq e_{2}\leq \ldots \leq e_{m+n}$, including *n* arrival points and *m* harvesting points. The time interval between two adjacent event points is called a *block/epoch*.

We consider a single user point-to-point transmission channel and make the same assumption as previous works that the transmitter can adaptively change its transmission rate *r*, which is related to its power *p*, through a function called rate-power  function. It is widely known that the rate-power function is convex and monotonous [5,16,27]. For example, in a AWGN (Additive White Gaussian Noise) channel, $r=\frac{1}{2}\log\left(1+p\right)$. In this paper, we use p=G(r) or $r=G^{-1}\left(p\right)$ to represent the general convex rate-power function.

We summarize notations used in this paper in Table 1 for readers to refer to.

### 3.2. Problem Formulation

We introduce an energy-efficient transmission problem where a transmitter needs to transmit its data, shared by multiple tasks, to the platform with the minimum completion time.

The transmitter can adaptively adjust its transmission rate to minimize transmission delay of all data requests according to the dynamic arrival of energy. The scheduling goal hence is to determine the rate scheduling policy.

**Definition** **1** **(rate** **scheduling** **policy).** *A rate scheduling policy is defined as the time-rate function r(t)≥0 which specifies the data transmission rate r(t) in time slot t, t=1,2,…,T, where T is the total time slots in consideration.*

The sensed data can be shared by multiple tasks as long as it fits in the time intervals of the tasks. A feasible schedule must satisfy the *task fulfillment constraint* that the data requirement of all tasks within their specified time period must be fulfilled. That it, task $J_{i}$ is satisfied as long as $w_{i}$ amount of sensed data is transmitted after time $a_{i}$,

(1) $$\begin{array}{cc} \sum_{a_{i}\leq t\leq T}r\left(t\right)\geq w_{i}, & \forall J_{i}\in J. \end{array}$$

Note that the data transmitted with rate r(t) in time slot *t* can be shared by (or equivalently be used to meet the requirement of) any task $J_{i}$ alive at time $t\in [a_{i},T]$.

A rate scheduling policy must satisfy the *energy causality constraints*, that is, the total depleted energy by time *t* should not exceed the total energy harvested,

(2) $$\begin{array}{cc} \sum_{1\leq \tau \leq t}G\left(r\left(\tau \right)\right)\leq \sum_{k:c_{k}\leq t}E_{k}, & \forall t\in [1,T]. \end{array}$$

**Definition** **2** **(min-T** **problem).** The completion time minimization problem is to minimize the transmission completion time T, under the task fulfillment constraints Equation (1) and the energy causality constraints Equation (2).

### 3.3. Overview of Our Solutions

For ease of reading, we introduce the overview of our solutions in this subsection.

It is natural to ask whether we can directly implement an algorithm with the goal of minimizing the transmission time. However, we were facing much difficulty, since the optimal transmission time is related with what rates are determined at each time slot, and even if we had known partial optimal rate allocation in some period, we cannot determine the minimum transmission time unless we have a complete figure about the optimal rate policy. Considering this challenge, we attempt to find a correct upper/lower bound of the optimal/minimum transmission time for the min-T problem, using which we can locate the optimal transmission time by developing searching strategies. Fortunately, we found that the optimal solution for the min-E problem (to be defined below) can act as such a role.

Define *E* as the *energy consumption* incurred by a rate schedule r(t), which is computed as

(3) $$E=\sum_{1\leq t\leq T}p\left(t\right)=\sum_{1\leq t\leq T}G\left(r\left(t\right)\right).$$

**Definition** **3** **(min-E** **problem).** Given a deadline T, the energy minimization problem is to (1) find the optimal solution to minimize the energy consumption E under the task fulfillment constraints Equation (1) and the energy causality constraints Equation (2), or (2) report if no feasible solution exists.

For the min-E problem, note that when the given deadline *T* is too early, there may be no feasible solution satisfying all constraints.

We note that computing the optimal solution for min-E problem still requires much effort to address the trade-off introduced by data sharing and energy harvesting, which is not addressed in prior works. In this work, we address this challenge by decomposing the problem into two sub-problems. Then we attempt to combine their solutions by iteratively comparing two rate curves of the sub-problems and merging them as a final correct curve of the optimal solution. Such a decomposition-based method is of its independent interest in solving complex rate scheduling problems, which has not been proposed in the literature, according to the knowledge of the authors.

Finally, taking such an intermediate solution as a building block, we try to figure out what is the optimal rate policy minimizing the transmission completion time.

In the following sections, we will first develop an optimal algorithm to determine the feasibility and output the optimal schedule for the min-E problem in Section 4, and then we will move on to solve the original min-T problem in Section 5.

## 4. Min-Energy Rate Schedule under a Given Deadline

In this section, we focus on the min-E  problem with a given deadline. We will first investigate some basic properties of the optimal solution. Then, we will decompose the min-E problem into two simplified models and figure out the relationship between the min-E problem and the decomposed problems. Finally, we develop an optimal algorithm to compute the optimal rate schedule for the min-E problem.

### 4.1. Basic Properties of Optimal Rate Schedule

Define the optimal rate scheduling policy for the min-E problem to be $r^{opt}\left(t\right)$ if it exists, which is referred to as $r^{opt}$ for short. We start by introducing some optimality properties about $r^{opt}\left(t\right)$ in the following lemmas.

Before we start, we first introduce the concept of *equalization* that will be used in our proofs. Given two rates $r_{1},r_{2}$, if we can equalize the two rates to $\frac{r_{1}+r_{2}}{2}$, the power consumption would decrease due to the fact that $2G\left(\frac{r_{1}+r_{2}}{2}\right)<G\left(r_{1}\right)+G\left(r_{2}\right)$ for convex rate-power functions. This method is called *equalization*.

We present the following two basic lemmas which can be easily extended from prior works that do not consider data sharing [16,20] (the detailed proof is omitted here).

**Lemma** **1.** $r^{opt}\left(t\right)$ changes only at event points.

**Lemma** **2.** $r^{opt}\left(t\right)$ is non-decreasing.

These two lemmas show that $r^{opt}\left(t\right)$ is a step/staircase function. In the following discussion, when we refer to a step,  we mean a unique and consecutive part of a step function with constant rate. Specifically, let $r_{i}$ be the transmission rate of step *i* in $r^{\mathrm{opt}}$. Accordingly, the ordered sequence of all the steps of a step function will be called a *step sequence*.

Then, we derive two properties of the optimal rate scheduling policy under the data sharing setting.

**Lemma** **3.** If $r^{opt}\left(t\right)$ increases at a harvesting point $c_{i}$ at which no task arrives, then the battery must be used up right before $c_{i}$. Viz., $\sum_{t=1}^{c_{i}-1}G\left(r^{opt}\left(t\right)\right)=\sum_{k:c_{k}<c_{i}}E_{k}$.

**Proof.**  We prove the lemma by contradiction. Suppose on the contrary, $r^{opt}\left(t\right)$ increases at a harvesting point $c_{i}$, but there remains some amount of energy at time slot $c_{i}-1$. We focus on interval $\left[c_{i}-1,c_{i}\right]$. Since there is no other task request arriving at $c_{i}$, it implies that if we moved a small amount of data from time slot $c_{i}$ to be transmitted at $c_{i}-1$, it would save some energy and would not violate any delay constraint, leading to a contradiction. This completes the proof. ☐

It is worth noticing that the condition that no task arrive at $c_{i}$ is necessary. Because otherwise if a task with a large workload also arrives at $c_{i}$, say $J_{i}=\left(c_{i},w_{i}\right)$, then we cannot move some data from time slot $c_{i}$ to $c_{i}-1$ since the delay constraint $\sum_{t=c_{i}}^{T}r\left(t\right)\geq w_{i}$ of this task may not hold any more.

**Lemma** **4.** If $r^{opt}\left(t\right)$ increases at an arrival point $a_{i}$ at which no energy harvesting occurs, then the total transmitted data from this point to the deadline T will be equal to the required data of task $J_{i}$. Viz., $\sum_{t=a_{i}}^{T}r^{opt}\left(t\right)=w_{i}$.

**Proof.**  First of all, we have $\sum_{t=a_{i}}^{T}r^{opt}\left(t\right)\geq w_{i}$, since the delay constraint of every task must be satisfied. Suppose $\sum_{t=a_{i}}^{T}r^{opt}\left(t\right)$ is strictly greater than $w_{i}$. Note that, we have $a_{1}<a_{2}<\ldots <a_{n}$. Moreover, $r^{opt}$ is non-decreasing according to Lemma 2. Hence, we can always find an epoch in $\left[a_{i},T\right]$ and equalize some small amount of data from that epoch to the epoch right before $a_{i}$ that has smaller rate than that one in $\left[a_{i},T\right]$, which would not violate the delay constraint of task $J_{i}$ since $\sum_{t=a_{i}}^{T}r^{opt}\left(t\right)>w_{i}$. Moreover, since no energy arrives at $a_{i}$, moving a small amount of energy used at $a_{i}$ to the time before it would not violate the energy causality constraint. This adjustment would save some energy by the convexity of the rate-power function, resulting in a contradiction to the optimality of $r^{opt}$. Thus, under the optimal policy, the delay constraint at that point must be satisfied as an equality. ☐

Lemmas 1–4 together show that $r^{opt}\left(t\right)$ is a non-decreasing step function that changes its rate either at a harvesting point or at an arrival point.

According to Lemmas 3 and 4, we have a direct corollary for the case that both a task request and a harvesting event occur simultaneously,

**Corollary** **1.** If $r^{opt}\left(t\right)$ increases at a point e at which both a task request $J_{i}=\left(e,w_{i}\right)$ and a harvesting event $H_{i}=\left(e,E_{i}\right)$ occur, then either the total transmitted data from e to T will be equal to $w_{i}$, or the battery is used up just right before time slot e.

### 4.2. Problem Decomposition

Although a deadline is given, the min-E problem is still complex with dynamic arrivals of both energy and requests. These arrival densities together have an impact on the allocation of transmission rate. Intuitively, an efficient rate schedule in an energy harvesting communication system tends to properly use partial energy early to avoid causing high density of remained energy in late periods (which is energy inefficient by the convexity of rate-power function). However, the efficient data sharing scheduling tends to reduce the traffic transmitted in early periods and increase data transmission in late periods so as to allow more data sharing.

Observing the above dilemma in dealing with the energy harvesting and data sharing, in this work, we address the challenge/trade-off by decomposing the problem into sub-problems. We then combine their solutions to form the optimal solution for the original problem. According to the best of our knowledge, no similar method has ever been proposed in the literature.

Note that previous Lemmas 3 and 4 present properties of the optimal increasing point in terms of energy harvesting and task requesting, respectively. This implies that we may decompose the problem into two simpler models: one is the transmission only with energy harvesting, and the other is the transmission only with task requests and data sharing. Thus, before deriving the structure of the optimal solution for the min-E problem, we will introduce these two simpler models.

We first introduce the DCRS problem that does not consider energy harvesting, as defined in Definition 4.

**Definition** **4.** Given a deadline T, the delay-constrained-only rate scheduling problem (DCRS problem) is to find a rate function such that the total energy consumption is minimized, subject to the delay constraints of all task requests described in Equation (1) under the data sharing setting.

For DCRS problem, Wu et al. [9,26], propose an optimal algorithm called Interval-Delete to search for the task with the largest average data density and then iteratively fix a part of the optimal rate function by deleting the corresponding time interval. We call the optimal rate function for DCRS problem the *ID rate schedule* and use $r^{ID}\left(t\right)$ to represent it (or $r^{ID}$ for short if there is no ambiguity).

Next, we introduce the EHRS problem that does not consider data requests and data sharing, as described in Definition 5.

**Definition** **5.** Given a deadline T, the energy-harvesting-only rate scheduling problem (EHRS problem) is to determine a rate schedule, such that the total transmitted data is maximized before the deadline T, subject to the energy causality constraints of Equation (2).

In contrast to DCRS problem, there is no concept of *data requests* or *data sharing*. It is assumed that there is enough data bits to be transmitted by the transmitter at the beginning of transmission, and the only objective is to send as much data bits as possible. For EHRS problem, an optimal algorithm that recursively fixes all parts of the optimal solution is provided in [28]. We call the optimal rate function for EHRS problem the *MT rate schedule* and use $r^{MT}\left(t\right)$ (or $r^{MT}$ for short) to represent it.

It has been proved in previous work that both $r^{ID}$ and $r^{MT}$ are non-decreasing step functions. Specifically, the increasing point of $r^{ID}$ must be a task arrival point and $r^{ID}$ follows a similar property as described in Lemma 4. Also, the increasing point of $r^{MT}$ must be corresponding to a harvesting event and it shares a property similar to Lemma 3.

For ease of presentation, we use $r_{i}^{ID}\left(t\right)$ or $r_{i}^{ID}$ for short (and correspondingly $r_{i}^{MT}\left(t\right)$ or $r_{i}^{MT})$ to denote the rate function of the *i*-th step of the step function $r^{ID}$ (and $r^{MT}$). We denote the step sequences of a step function r(t) as $S=\left\{S_{1},S_{2},\ldots \right\}$, where a triple $S_{i}=\left(r_{i},t_{i},l_{i}\right)$ is used to describe the *i*-th step of r(t), which means the *i*-th step with transmission rate $r_{i}$ starts at time slot $t_{i}$ and lasts for $l_{i}$ time slots (including time slot $t_{i}$). Thus, the end point of the *i*-th step is $t_{i}+l_{i}-1$. Specifically, we use $S^{ID}=\left\{S_{1}^{ID},S_{2}^{ID},\ldots \right\}$ and $S^{MT}=\left\{S_{1}^{MT},S_{2}^{MT},\ldots \right\}$ to represent the step sequences of $r^{ID}$ and $r^{MT}$ respectively, where $S_{i}^{ID}=\left(r_{i}^{ID},t_{i}^{ID},l_{i}^{ID}\right)$ and $S_{i}^{MT}=\left(r_{i}^{MT},t_{i}^{MT},l_{i}^{MT}\right)$.

**Lemma** **5.** If $r_{1}^{ID}>0$, then $J_{1}$ has the largest workload among all the tasks. That is, $w_{1}=\max_{i:J_{i}\in J}\left\{w_{i}\right\}$.

**Proof.**  It can be proved by contradiction easily. Suppose $J_{1}$ is not the request with the largest workload. We can pick the one with largest required data, say $J_{k}\left(k\neq 1\right)$, then it is obvious that $J_{1}$ can completely share the data of $J_{k}$, which means there is no need to allocate a rate larger than 0 with $r_{1}^{ID}>0$ until $J_{k}$ arrives. This leads to a contradiction and proves the lemma. ☐

Note that the same observation as the lemma above is also applicable to the optimal solution $r^{opt}$ of the min-E problem.

### 4.3. The Bottleneck-Select Algorithm

After introducing the basic properties of $r^{opt}$ and the two decomposed simple sub-problems, we are ready to examine the key properties of the min-E problem that would guide the design of our algorithm.

On one hand, if the energy is sufficient (or more precisely, if for all time slots t∈[1,T], harvested energy is sufficient to support $r^{ID}$), then we have $r^{opt}=r^{ID}$, since $r^{ID}$ is the optimal rate schedule that achieves the minimum energy consumption given a deadline *T*. On the other hand, if harvested energy is insufficient to support $r^{ID}$, the rate level must be decreased in order to avoid energy shortage. However, if the rate level is lowered down too much, then less data would be transmitted in the current epoch, which would lead to a situation that more data will be transmitted later with higher rate which is energy inefficient. Thus, we hope to reach a good trade-off between the amount of transmitted data and energy consumption, and allocate proper transmission rate to overcome the energy shortage.

Our high level idea is to compare the rates of $r^{ID}$ and $r^{MT}$ to help figure out what rate the optimal solution $r^{opt}$ should choose.

Theorems 1 and 2 below together show the key properties that would help determine the rate.

**Theorem** **1.** If $r_{1}^{ID}\geq r_{1}^{MT}$, then the optimal solution $r^{opt}$ for the min-E problem exactly equals to $S_{1}^{MT}$ during interval $\left[1,l_{1}^{MT}\right]$.

**Proof.**  We prove Theorem 1 by contradiction. If $r^{opt}$ is not equal to $S_{1}^{MT}$ under the condition that $r_{1}^{ID}\geq r_{1}^{MT}$ in interval $\left[1,l_{1}^{MT}\right]$, then we consider all the possible relationships between $r^{opt}$ and $r^{MT}$ during interval $\left[1,l_{1}^{MT}\right]$ one by one:

(1) $r^{opt}\left(t\right)>r_{1}^{MT}$ for all *t* in $\left[1,l_{1}^{MT}\right]$.

According to the properties of $r^{MT}\left(t\right)$, energy will be used up by time slot $l_{1}^{MT}$, thus $r^{opt}$ cannot be supported to have larger rate in the whole interval $\left[1,l_{1}^{MT}\right]$. Therefore such a case is impossible to occur.

(2) The curve of $r^{opt}\left(t\right)$ intersects with that of $r^{MT}$ in $\left[1,l_{1}^{MT}\right]$.

**Figure 2:**
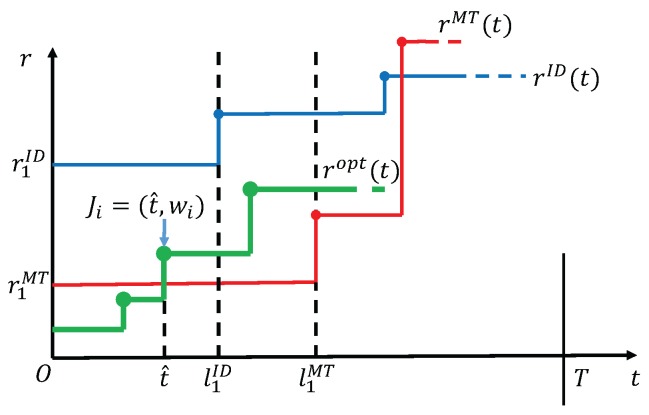
The case that $r^{opt}$ intersects with $r^{MT}$.

An examplary diagram corresponding to this case is shown in Figure 2. By the non-decreasing property of $r^{opt}$ in Lemma 2, it is a fact that there is at most one intersection between $r^{opt}$ and $r^{MT}$ in interval $\left[1,l_{1}^{MT}\right]$. Let the corresponding time slot of the intersection be $\hat{t}$. Then $r^{opt}\left(t\right)\leq r_{1}^{MT}$ in $\left[1,\hat{t}-1\right]$ and $r^{opt}\left(t\right)\geq r_{1}^{MT}$ in $\left[\hat{t},l_{1}^{MT}\right]$. We claim that $\hat{t}$ cannot be a harvesting point, because otherwise according to Lemma 3 energy is used up by time $\hat{t}$, which contradicts the feasibility of $r^{MT}$ that has a larger rate than $r^{opt}$ by time $\hat{t}$. Thus, $\hat{t}$ can only be an arrival point. Let the arrival task at $\hat{t}$ be $J_{i}=\left(\hat{t},w_{i}\right)$. Now that $\hat{t}$ is an arrival point that $r^{opt}$ increases, we have $\sum_{\tau =\hat{t}}^{T}r^{opt}\left(\tau \right)=w_{i}$ according to Lemma 4. Meanwhile, for $r^{ID}$, it must satisfy $\sum_{\tau =\hat{t}}^{T}r^{ID}\left(\tau \right)\geq w_{i}$ to follow the delay constraint of task $J_{i}$. In addition, since $r_{1}^{ID}\geq r_{1}^{MT}>0$, which means that the first task $J_{1}=\left(1,w_{1}\right)$ has the largest amount of data request among all tasks according to Lemma 5. So $\sum_{t=1}^{T}r^{ID}\left(t\right)=w_{1}$ and at least $\sum_{t=1}^{T}r^{opt}\left(t\right)\geq w_{1}$. Then,

(4) $$\begin{array}{c} \sum_{\tau =1}^{\hat{t}-1}r^{ID}\left(\tau \right)=\sum_{\tau =1}^{T}r^{ID}\left(\tau \right)-\sum_{\tau =\hat{t}}^{T}r^{ID}\left(\tau \right)\leq w_{1}-w_{i}, \end{array}$$

(5) $$\begin{array}{c} \sum_{\tau =1}^{\hat{t}-1}r^{opt}\left(\tau \right)=\sum_{\tau =1}^{T}r^{opt}\left(\tau \right)-\sum_{\tau =\hat{t}}^{T}r^{opt}\left(\tau \right)\geq w_{1}-w_{i}. \end{array}$$

Combining Equations (4) and (5), we have $\sum_{\tau =1}^{\hat{t}-1}r^{opt}\left(\tau \right)\geq \sum_{\tau =1}^{\hat{t}-1}r^{ID}\left(\tau \right)$. However, it is clear that $\sum_{\tau =1}^{\hat{t}-1}r^{opt}\left(\tau \right)<\sum_{\tau =1}^{\hat{t}-1}r^{ID}\left(\tau \right)$ according to the precondition that $r^{opt}\left(t\right)\leq r_{1}^{ID}$ in interval $\left[1,\hat{t}-1\right]$, which brings us a contradiction. Thus, such a case is also impossible to occur.

(3) $r^{opt}\left(t\right)<r_{1}^{MT}$ in $\left[1,l_{1}^{MT}\right]$.

We consider two sub-cases: one is that $r^{opt}\left(t\right)$ is not constant in $\left[1,l_{1}^{MT}\right]$, the other is the constant case. For the former, we can follow the discussion similar to the proof of case (2) above, except that the intersection point in the discussion becomes the first point at which $r^{opt}\left(t\right)$ increases, thus we omit the details. For the latter, we extend the interval $\left[1,l_{1}^{MT}\right]$ and can always find the first point at which $r^{opt}\left(t\right)$ increases ($r^{opt}\left(t\right)$ cannot keep to be a constant rate during the whole transmission by time *T*, otherwise it will contradict the existence of $r^{ID}$, because the delay constraint will be violated). Let the first increasing point of $r^{opt}$ be a time $\hat{t}$ with $\hat{t}>l_{1}^{MT}$, then $\hat{t}$ must be a task arrival point or an energy harvesting point. On one hand, if $\hat{t}$ is an energy harvesting point, then energy is used up by $\hat{t}$, which implies a contradiction since $r_{1}^{MT}$ with a larger rate than $r^{opt}$ cannot be supported in $\left[1,\hat{t}\right]$. On the other hand, if $\hat{t}$ is an arrival point, then following the same proof as that of case (2) can also deduce a contradiction. These together would remove the possibilities of the case.

In summary, $r^{opt}\left(t\right)$ must be equal to $r_{1}^{MT}$ in interval $\left[1,l_{1}^{MT}\right]$. ☐

Symmetrically, we have the following theorem, where the detailed proof is moved to Appendix A.

**Theorem** **2.** If $r_{1}^{ID}<r_{1}^{MT}$, then the optimal solution $r^{opt}\left(t\right)$ for min-E problem exactly equals to $S_{1}^{ID}$ during interval $\left[1,l_{1}^{ID}\right]$.

Based on Theorems 1 and 2, we are able to fix the rate schedule $r^{opt}\left(t\right)$ in interval $\left[1,l_{1}^{MT}\right]$ or $\left[1,l_{1}^{ID}\right]$. Then, starting with the next new time slot, the same problem would repeat, if we could correctly update the sets of tasks and harvesting events, until all the tasks are finished.

First, we introduce the update module, whose function is to generate the same smaller-size problem after a part of the rate schedule $r^{opt}$ is fixed. Let the rate and corresponding interval of the fixed part in $r^{opt}$ be *r* and [1,l], respectively. Since a part of the optimal solution has been fixed, we *shift*  the time axis by *l* time slots and properly update the tasks and harvestings that arrive within and after the time duration of the fixed part by treating them as new instances. The detailed implementation is presented in Algorithm 1.

**Algorithm 1** Update(J,H,r,l)
1:update the deadline *T* to be T−l.2:for each task $J_{i}$ with $a_{i}\leq l$, update its arrival time to be $a_{i}=1$ and the remaining workload to be $\max\{w_{i}-r\cdot \left(l-a_{i}+1\right),0\}$.3:among all the tasks with $a_{i}=1$, reserve the task with largest workload and remove all the others.4:for each task $J_{i}$ with $a_{i}>l$, update its arrival time to be $a_{i}-l$.5:let $E_{new}=\sum_{c_{i}\leq l}E_{i}-G\left(r\right)\cdot l$, remove all the harvestings with $c_{i}\leq l$.6:create a new harvesting, let its arrival time be 1 and amount of energy be $E_{new}$.7:for each harvesting $H_{i}$ with $c_{i}>l$, update its arrival time to be $c_{i}-l$.


Then, we present the final algorithm for computing the optimal schedule $r^{opt}$ of the min-E problem. The idea is to compare the first steps of rates $r^{ID}$ and $r^{MT}$ in the two decomposed problems to find the bottleneck. If $r_{1}^{ID}\geq r_{1}^{MT}$, we select $S_{1}^{MT}$ as the first part of $r^{opt}$, otherwise, we select $S_{1}^{ID}$. After fixing the first part, we recursively update the problem and compute the residual part of $r^{opt}$. The detailed implementation is presented in Algorithm Bottleneck-Select.

It is worth noticing that the min-E problem with a given deadline may have no feasible solutions. This happens if the harvested energy is insufficient, or the deadline is set to be too early so that some delay constraints in Equation (1) are impossible to be met. To detect the infeasibility of the input case, we just need to check whether there exists some task that has not been finished at the end of the while loop, as implemented in Line 15 in Algorithm 2.

Finally, we conclude that Algorithm Bottleneck-Select either returns the optimal solution or identifies the infeasibility for the min-E problem.

**Algorithm 2** Bottleneck-Select (J,H,T)
1:let r(t)←0 in [1,T], t←0.2:**while** t≤T **do**3: compute the first step of *ID rate schedule* $S_{1}^{ID}=\left(r_{1}^{ID},t_{1}^{ID},l_{1}^{ID}\right)$.4: compute the first step of *MT rate schedule* $S_{1}^{MT}=\left(r_{1}^{MT},t_{1}^{MT},l_{1}^{MT}\right)$.5: **if** $r_{1}^{ID}\geq r_{1}^{MT}$ **then**6:  $r\left(t\right)\leftarrow r_{1}^{MT}$ in $\left[t+1,t+l_{1}^{MT}\right]$.7:  Update($J,H,r_{1}^{MT},l_{1}^{MT}$).8:  $t\leftarrow t+l_{1}^{MT}$.9: **else**10:  $r\left(t\right)\leftarrow r_{1}^{ID}$ in $\left[t+1,t+l_{1}^{ID}\right]$.11:  Update($J,H,r_{1}^{ID},l_{1}^{ID}$).12:  $t\leftarrow t+l_{1}^{ID}$.13: **end if**14:**end while**15:**if** there exists some task that has not been finished **then**16: **return** infeasible17:**end if**18:**return** r(t)

**Theorem** **3.** Algorithm Bottleneck-Select computes the optimal rate schedule for the min-E problem when a feasible schedule exists, and determines the infeasibility of the input otherwise, in $O(\left(n+m\right)\left(n^{2}+m\right))$ time.

**Proof.**  We prove the optimality for minimizing the energy consumption by induction on iterations. In the first iteration, Algorithm Bottleneck-Select correctly computes and fixes the partial optimal schedule that minimizes the energy consumption by Theorems 1 and 2, which serves as the induction basis. Suppose Algorithm Bottleneck-Select fixes the optimal rate allocation in interval $\left[1,l^{\left(k\right)}\right]$ after the first *k* iterations (k≥1), we need to prove that this property also holds after the (k+1)-th iteration. At the beginning of the (k+1)-th iteration, all tasks with $a_{i}\leq l^{\left(k\right)}$ are updated by the Update operation in the *k*-th iteration. Specifically, each task with $a_{i}\leq l^{\left(k\right)}$ has $\max\{w_{i}-\sum_{t=a_{i}}^{l^{\left(k\right)}}r\left(t\right),0\}$ workload to be finished in $\left[l^{\left(k\right)}+1,T\right]$, and among them only the task with the largest remaining workload is retained and regarded as a new task at slot $l^{\left(k\right)}+1$, according to the sharing nature of the data. This operation ensures that no extra workload is dealt with later. Then, in the (k+1)-th iteration, it can be verified that transmitting with the computed rate $r_{1}^{MT}$ (or $r_{1}^{ID}$) is energy-optimal for the new task set and harvesting event set by similarly applying the proof of Theorems 1 and 2 to the updated instance. Finally, when the iteration terminates with t>T, according to the correctness of hypothesis and inductions above, Algorithm Bottleneck-Select has fixed the optimal min-energy rate schedule in [1,T].

Next, we analyze the computational complexity. The *while*   loop repeats at most (n+m) times, since there are totally (n+m) event points and at least one event point is reached in each loop. To compute $S_{1}^{MT}$, we only need to scan the set of harvesting events once in at most O(m) time. However, we must construct the whole rate schedule of $r^{ID}$ before we obtain $S_{1}^{ID}$, because Algorithm Interval-Delete in [26] partially fixes $r^{ID}$ in a back-to-front manner, which takes at most $O(n^{2})$ time. The Update part works with O(n+m) time and is not time consuming. Therefore, the total time complexity is $O(\left(n+m\right)\cdot \left(n^{2}+m\right))$. ☐

## 5. Optimal Rate Schedule for Min-T Problem

After we have solved the min-E problem with a given deadline, we move forward to solve the min-T  problem. The difference is that now the deadline, or the overall transmission completion time, becomes a variable we need to optimize.

Let $r^{*}\left(t\right)$ and $T^{*}$ be the rate allocation and the corresponding completion time of the optimal solution of the min-T problem, respectively. We start again by deriving the properties of the optimal policy, as shown in the following lemmas.

**Lemma** **6.** Under the optimal policy, all the harvested energy must be used up by the end of transmission.

This lemma can be easily established. Because if it is not the case, we can always use the remaining energy to increase the rate of some former epoch and shorten the transmission completion time.

Moreover, by extending the properties of Lemmas 1–4, we can easily have the following properties for the optimal solution of the min-T problem.

**Lemma** **7.** The optimal rate function $r^{*}\left(t\right)$ of the min-T problem satisfies,

- $r^{*}\left(t\right)$ is a non-decreasing step function and only changes the rate at harvesting point or arrival point.
- If $r^{*}\left(t\right)$ increases only at an arrival point, then the total transmitted data from this point to the end of the transmission will be equal to the data required at this point;
- if $r^{*}\left(t\right)$ increases only at a harvesting point, then the battery must be used up just right before this point;
- if $r^{*}\left(t\right)$ increases at a point e at which both a task $J_{i}=\left(e,w_{i}\right)$ and a harvesting event $H_{i}=\left(e,E_{i}\right)$ occur, then either the total transmitted data from this point to the end of transmission will be equal to $w_{i}$, or the battery is used up just right before e.

Although the min-T problem is quite different from the min-energy problem, the structure of their optimal solutions are closely associated. As shown in the following lemma, we could yield the same optimal rate schedule of the min-T problem and that of the min-E problem, under condition that we know the optimal completion time $T^{*}$ beforehand,

**Lemma** **8.** Under the same task set J and the set of energy harvesting events H, the min-T problem and min-E problem yield the same rate schedule if the deadline in the min-E problem is set to be exactly the minimum transmission time $T^{*}$ of the min-T problem.

**Proof.**  We prove by contradiction. Let $r^{opt}\left(t\right)$ and $r^{*}\left(t\right)$ be the optimal rate scheduling policy of the min-E problem and min-T problem, respectively. Assume that the deadline of the min- problem is set to be exactly the shortest transmission completion time of the min-T problem, that is, $T=T^{*}$, but $r^{opt}\left(t\right)\neq r^{*}\left(t\right)$. Since $r^{opt}\left(t\right)$ yields the minimum energy consumption among all the feasible solutions, it implies that if we replace $r^{*}\left(t\right)$ with $r^{opt}\left(t\right)$, some energy might be saved and then this amount of energy can be used to shorten the transmission completion time, which contradicts the optimality of $T^{*}$. ☐

More importantly, we can easily have the following key lemma to help design our algorithm for the min-T problem based on the results above.

**Lemma** **9.** Under the same task set J and the set of energy harvesting events H, if the deadline of the min-E problem is set to be $T\geq T^{*}$, then there exists a feasible solution for the min-E problem, otherwise there is no feasible solution.

Lemmas 8 and 9   imply that, by checking the feasibility of the min-E problem given a completion time, we can determine whether such a completion time to be returned by a schedule of the min-T problem is good enough or not. Thus, we can design some search strategy to determine the unique optimal transmission time in the min-T problem. Generally, the high level idea of our strategy can be divided into two phases: we first estimate a good lower bound $T_{lb}$ and upper bound $T_{ub}$ of $T^{*}$ by a doubling strategy (called *estimation phase*),  then we apply binary search to exactly determine the minimum completion time $T^{*}$ precisely (called *determination phase*).

The details are as follows. For the estimation phase, we properly guess an end point (deadline) of transmission and run Bottleneck-Select to test whether the given deadline is too early. If it is the case, we double the deadline and test again, until we reach a case that all the tasks can be done before the deadline. Then, that deadline is an upper bound of the optimal completion time $T^{*}$, and the deadline guessed right before that one is set to be the lower bound of $T^{*}$. The detailed description is shown in Algorithm Estimate. Note that in the first line of Algorithm 3, $a_{n}$ denotes the arrival time of the last task, which is a good lower bound of $T^{*}$ to be set at the beginning.

**Algorithm 3** Estimate (J,H)1:let $T_{lb}\leftarrow a_{n}$, $T_{ub}\leftarrow a_{n}+1$.2:set sign←false.3:**while** sign is not true **do**4: run Bottleneck-Select($J,H,T_{ub}$) to determine if all the tasks can be finished before $T_{ub}$, if it is, set sign←true, otherwise, set $T_{lb}=T_{ub}$, and $T_{ub}\leftarrow T_{ub}\times 2$.5:**end while**6:**return** $T_{lb}$, $T_{ub}$


For the determination phrase, Algorithm 4 Locate is developed to determine the optimal transmission time precisely. The idea is that, starting with the interval $\left[T_{lb},T_{ub}\right]$ returned by Estimate, we test a mid point, say $T_{mid}$, to detertime the optimal completion time by running Bottleneck-Select over that point (as a given deadline) and checking its feasibility. If Bottleneck-Select returns a feasible solution, then we continue to search in interval $\left[T_{lb},T_{mid}\right]$. Otherwise, we continue to search in interval $\left[T_{mid},T_{ub}\right]$.

**Algorithm 4** Locate ($J,H,T_{lb},T_{ub}$)
1:initialize r(t)←0.2:**while** $T_{lb}+1<T_{ub}$ **do**3: let $T_{mid}\leftarrow \frac{T_{lb}+T_{ub}}{2}$.4: r(t)← Bottleneck-Select($J,H,T_{mid}$), record sign←true if there is a feasible solution, otherwise record sign←false.5: **if** sign is true **then**6:  $T_{ub}\leftarrow T_{mid}$.7: **else**8:  $T_{lb}\leftarrow T_{mid}$.9: **end if**10:**end while**11:r(t)← Bottleneck-Select($J,H,T_{lb}$).12:**if** r(t) is a feasible solution **then**13: **return** $T_{lb}$, r(t).14:**else**15: r(t)← Bottleneck-Select($J,H,T_{ub}$)16: **return** $T_{ub}$, r(t).17:**end if**


Finally, we conclude the optimality of the proposed algorithm in the following theorem.

**Theorem** **4.** *Algorithm* Estimate *and* Locate together compute the optimal rate schedule for the min-T problem in $O(\log T^{*}\cdot \left(n+m\right)\cdot \left(n^{2}+m\right))$ time.

**Proof.**  First, it is obvious that Estimate correctly returns a lower bound and an upper bound of $T^{*}$ based on Lemma 9, and the optimality of Algorithm Locate can be proved based on the *binary search rule*. Then, it is easy to see that both Estimate and Locate call Bottleneck-Select $O(\log T^{*})$ times during the estimation and determination phrases. Therefore, the total time complexity of the two algorithms is $O(\log T^{*}\cdot \left(n+m\right)\cdot \left(n^{2}+m\right))$. ☐

## 6. Online Rate Schedule

In this section, we study the online min-T problem to minimize the transmission completion time without any prior knowledge of task requests and harvesting events.

Applying the properties of the optimal offline rate allocation function, we propose an online Algorithm Online-Select, which works in an *event-driven* manner. It transmits at a constant rate level based on currently known information until a new event (a task request or a harvesting) occurs, and tries to share data as much as possible. The basic mechanism is, at each time slot *t*, we keep a rate level that minimizes the transmission completion time of all the arrived task requests so far, with currently harvested available energy.

As time goes by, when a harvesting event occurs, the energy is added to the battery. When a task request comes, we check whether its required workload is larger than the current total demanded workload. If this is true, we update the total demanded workload to be the required data in order to ensure the fulfillment of the new task, otherwise it is unnecessary to increase the demand workload, since this task can share data with previous ones. Then we allocate the transmission rate according to the updated workload and available energy, by solving the equations described in Line 16 in Algorithm 5. Generally, on one hand, if the available energy is sufficient, the rate will increase so as to shorten the transmission time of current workload. On the other hand, if the requests are intensive, the rate will decrease and the transmission time will be lengthened in order to overcome energy shortage.

**Algorithm 5** Online-Select (J,H)1:initialize r(0)←0, t←1, E←0 be the current available energy, D←0 be the current demanded workloads that need to be done.2:**while** time goes by and J has not been finished **do**3: **if** there is no event occuring at time slot *t* **then**4:  **if** there remains some workloads **then**5:   r(t)←r(t−1)6:  **else**7:   r(t)←08:  **end if**9: **else**10:  **if** a task request $J_{i}=\left(a_{i},w_{i}\right)$ arrives at *t* **then**11:   $D\leftarrow \max(D,w_{i})$12:  **end if**13:  **if** a harvesting event $H_{i}=\left(c_{i},E_{i}\right)$ occurs at *t* **then**14:   $E\leftarrow E+E_{i}$15:  **end if**16:  r,τ← solve $\left\{\begin{array}{c} r\cdot \tau =D,\\ G(r)\cdot \tau =E. \end{array}\right.$17:  r(t)←r18: **end if**19: D←D−r(t)·120: E←E−G(r(t))·121: t←t+122:**end while**


## 7. Simulations

We have proved the optimality of the proposed algorithm for min-T problem in the offline setting. In this section, we further conduct simulations to show the performance of the online algorithm Online-Select.

We will compare our proposed Online-Select algorithm with the optimal offline solution and the three baselines, which are listed as follows.

- **OPT**, which is the optimal offline solution returned by the optimal algorithm developed in this work.
- **OPT without sharing**, which is the optimal offline solution of a variant of the min-T problem that does not consider data sharing [16].
- **Jing’s algorithm with sharing**, which is an offline algorithm in [16] that is adopted to work in the data sharing scenario by keeping its rate policy unchanged.
- **Online-Select without sharing**, which is a slightly modified version of our online algorithm Online-Select by simply adding new arrived workloads into the data buffer and transmitting with local optimal rate.

We implement the simulations by MATLAB. The simulation setting is as follows. The rate-power function modeling the AWGN channel is set to be $r=G^{-1}\left(p\right)=\frac{1}{2}\log\left(1+p\right)$, where *p* is in milliwatts (mW) and *r* is in kilobits per second (kpbs). Task arrival time $a_{i}$ is assumed to be a random integer that obeys uniform distribution U(1,300). The size of requested workload is assumed to follow normal distribution $N(450\;\mathrm{kb},{\left(100\;\mathrm{kb}\right)}^{2})$ by default. We also assume that the harvesting event arrives randomly following uniform distribution U(1,500), and the size of energy harvested is distributed uniformly in U(0.5h,50h) where the default value of *h* is 1000 mJ. In addition, both the number of tasks and harvestings are set to be 25 if not specified. Each point in the following figures is a mean value of 100 random instances. For ease of reading, the settings of the main parameters of the simulations are summarized in Table 2.

**Figure 3:**
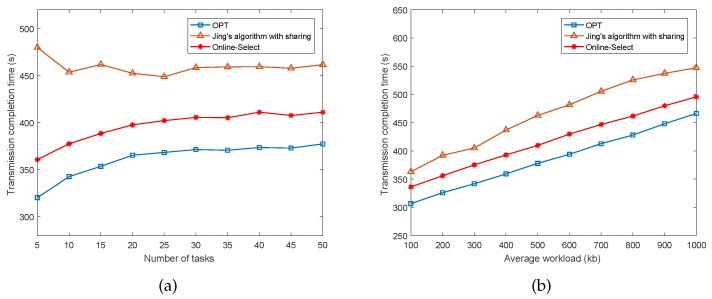
Performance of Online-Select as the requests change.

In Figure 3, we evaluate the performance of the algorithm as the number of tasks and average workload of tasks increase, respectively. The results are shown in Figure 3a,b. We can observe that in both cases, the curves of OPT and Online-Select increase as the number of tasks or the average workload of tasks increases, and they outperform that of *Jing’s algorithm with sharing*. Furthermore, the minimum completion time achieved by the online algorithm is within 1.2 times of the optimal solution.

**Figure 4:**
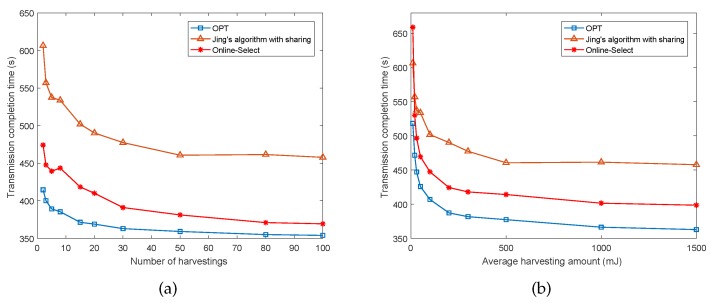
Performance of Online-Select as the harvestings change.

Next, we further evaluate the performance as the number of harvestings and amount of average harvested energy increase. Figure 4a,b demonstrate the results. We can see from the figure that the transmission completion time decreases when the number of harvestings or average amount of harvested energy increases. In both of the two sub-figures, both the solutions of our offline algorithm and online algorithm Online-Select outperform those of *Jing’s algorithm with sharing*, and the ratios between Online-Select and OPT are bounded within a factor of 1.3.

Last, we examine the effect of exploiting data sharing by comparing our solutions with two baselines that have not considered the data sharing among requests.

**Figure 5:**
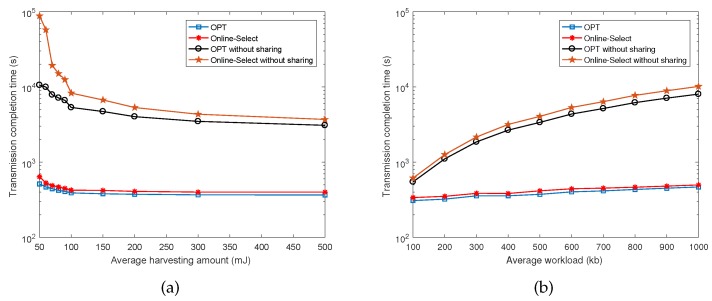
Effect of exploiting data sharing as the average harvesting amount/workload changes.

Figure 5a,b respectively demonstrate the results in terms of the change of average harvesting amount and average workload. It can be seen from the figures that the output of our online algorithm Online-Select is close to that of the optimal offline algorithm in both scenarios. It is also obviously that our offline optimal algorithm and online algorithm Online-Select significantly shorten the transmission completion time by exploiting data sharing, compared with baselines without data sharing.

Therefore, the simulations above validate the effectiveness of our algorithms.

## 8. Conclusions

This paper attempts to exploit the data sharing to enhance the energy utilization efficiency of energy harvesting wireless devices in data transmission. We formulate the problem as a completion time minimization problem while satisfying the data requests and the energy constraints under dynamic arrivals. For the offline scenario, we provide the optimal algorithm to minimize the transmission completion time. We also propose an efficient online algorithm with performance validated in simulations. Simulation results have validated that it significantly improves the completion time of the transmission under dynamic energy arrivals by exploiting the data sharing. One of our future work is to extend the work by considering the transmission in fading channel and the possible battery overflow during the transmission. We believe the decomposition method developed in this work is promising to be applied to solve more complex problems in designing rate scheduling policies.

## Figures and Tables

**Table 1:** Notations.

Symbol	Semantics
J	task set
$J_{i}$	*i*th task
$a_{i}$	arrival time of $J_{i}$
$w_{i}$	amount of data requested by $J_{i}$
H	the set of harvesting events
$H_{i}$	*i*th harvesting
$c_{i}$	harvesting time of $H_{i}$
$E_{i}$	amount of energy harvested by $H_{i}$
p=G(r)	rate-power function, the power consumed to achieve a rate *r*
*T*	transmission completion time
r(t)	data rate specified in time *t*
$r^{*}\left(t\right)$	optimal rate function for the min-T problem
$r^{opt}\left(t\right)$	optimal rate function for the min-E problem

**Table 2:** Settings of simulations.

Parameter	Meaning	Setting
*n*	the number of tasks	by default n=25
$a_{i}$	arrival time of the *i*-th task	random integer that obeys the uniform distribution of U(1,300)
$w_{i}$	workload of the *i*-th task	follows the normal distribution of $N(450\;\mathrm{kb},{\left(100\;\mathrm{kb}\right)}^{2})$
*m*	the number of harvestings	by default m=25
$c_{i}$	harvesting time of $H_{i}$	random integer that obeys the uniform distribution of U(1,500)
$E_{i}$	amount of harvested energy by $H_{i}$	follows the uniform distribution U(0.5h,50h), where *h* is 1000 mJ

## Appendix A. Proof of Theorem 2

We prove Theorem 2 through contradiction. Assume that $r^{opt}\left(t\right)$ does not equal to $S_{1}^{ID}$ under the condition that $r_{1}^{ID}<r_{1}^{MT}$, then we discuss all the possible relationships between $r^{opt}$ and $r^{ID}$ during interval $\left[1,l_{1}^{ID}\right]$ respectively.

Since $l_{1}^{ID}+1$ is the first increasing point of $r^{ID}$, $l_{1}^{ID}+1$ should be an arrival point and the total transmitted data from $l_{1}^{ID}+1$ to deadline *T* exactly equals to the required data of the arrival task, according to the fact that $r^{ID}$ shares a property similar to Lemma 4. Let the arrival task be $J_{i}=\left(l_{1}^{ID}+1,w_{i}\right)$, then we have $\sum_{t=l_{1}^{ID}+1}^{T}r^{ID}\left(t\right)=w_{i}$.

(1) $r^{opt}\left(t\right)>r_{1}^{ID}$ for all $t\in [1,l_{1}^{ID}]$

Since $r^{opt}\left(t\right)>0$, it is not hard to speculate that the first task $J_{1}=\left(1,w_{1}\right)$ requires the largest amount of data among all tasks. Thus, we have $\sum_{t=1}^{T}r^{opt}\left(t\right)=\sum_{t=1}^{T}r^{ID}\left(t\right)=w_{1}$. Due to the precondition that $r^{opt}\left(t\right)>r_{1}^{ID}$ for all $t\in [1,l_{1}^{ID}]$, we must have $\sum_{t=1}^{l_{1}^{ID}}r^{opt}\left(t\right)>\sum_{t=1}^{l_{1}^{ID}}r^{ID}\left(t\right)$. Then, we calculate the amount of transmitted data of $r^{opt}\left(t\right)$ in interval $\left[l_{1}^{ID}+1,T\right]$:

(A1) $$\begin{array}{cc} \sum_{t=l_{1}^{ID}+1}^{T}r^{opt}\left(t\right) & =\sum_{t=1}^{T}r^{opt}\left(t\right)-\sum_{t=1}^{l_{1}^{ID}}r^{opt}\left(t\right)\\ & <\sum_{t=1}^{T}r^{ID}\left(t\right)-\sum_{t=1}^{l_{1}^{ID}}r^{ID}\left(t\right)\\ & =\sum_{t=l_{1}^{ID}+1}^{T}r^{ID}\left(t\right)\\ & =w_{i}. \end{array}$$

This conflicts with the satisfaction of the delay constraint for task $J_{i}$, thus removes the possibility of the case under consideration.

(2) The curve of $r^{opt}$ intersects with the curve of $r^{ID}$ in interval $\left[1,l_{1}^{ID}\right]$.

By the non-decreasing property of $r^{opt}$, there exists only a single intersection in such case. We assume the corresponding time of the intersection is $\hat{t}$. We can derive that $\hat{t}$ cannot be a harvesting point. Otherwise, according to Lemma 3, energy is used up by time $\hat{t}$. However, because $r^{opt}\left(t\right)\leq r^{ID}\left(t\right)<r_{1}^{MT}$ for all $t\in [1,\hat{t}-1]$, it implies that $r_{1}^{MT}$ cannot be supported by the harvested energy, which is a contradiction. So $\hat{t}$ must be an arrival point. Then following the similar method described in the proof of Theorem 1 (case (2)), we can also deduce a contradiction.

(3) $r^{opt}\left(t\right)<r_{1}^{ID}$ for all $t\in [1,l_{1}^{ID}]$.

The proof is the same as case (2) except that the time $\hat{t}$ to be examined is not set to be the intersection but the first increasing point of $r^{opt}\left(t\right)$. The details are thus omitted here.

Based on all the discussions above, $r^{opt}\left(t\right)$ must be equal to $r_{1}^{ID}$ in interval $\left[1,l_{1}^{ID}\right]$.
